# Examining the relationship between reproductive empowerment and contraceptive self-injection: Tackling the endogeneity problem

**DOI:** 10.1371/journal.pone.0319330

**Published:** 2025-02-24

**Authors:** Megan M Lydon, Holly M Burke, Katherine M Anfinson, Tihut Mulugeta, Aderaw Anteneh, Teferi Teklu, Mario Chen

**Affiliations:** 1 FHI 360, Durham, North Carolina, United States of America; 2 PSI, Addis Ababa, Ethiopia; Icahn School of Medicine at Mount Sinai Department of Pharmacological Sciences, UNITED STATES OF AMERICA

## Abstract

**Background:**

Self-care interventions, including contraceptive self-injectables such as subcutaneous depot medroxyprogesterone acetate (DMPA-SC), are hypothesized to be empowering to users. It is also believed that those who are empowered are more likely to use self-care. Though critical for ensuring equity of these interventions, evidence for the relationship between empowerment and contraceptive self-care is scant. However, studying this relationship is challenging. In addition to the potential reversed causality between these two constructs, empowerment is determined by similar factors as the motivation for using self-care, contributing to an endogeneity problem. If not addressed, endogeneity can lead to incorrect causal assertions.

**Methods:**

Using data from a study of 400 women in Addis Ababa, Ethiopia who opted to self-inject DMPA-SC, we assessed the directionality between the two constructs. First, we assessed the change in empowerment after participants’ first self-injection. Second, we assessed the effect of empowerment on potential future use of self-injection. To address potential endogeneity, we identified instrumental variables of empowerment and then applied a two-stage regression approach to predict desire to continue self-injecting at follow-up with an instrument for empowerment, controlling for other variables.

**Results:**

Empowerment scores among the 343 women who were followed-up were high and did not significantly change from baseline to endline. Most women (78%) wanted to continue self-injecting. The following variables were identified and used as instruments: religion, employment status and post-secondary school attendance. The final model did not identify a significant relationship between desire to continue self-injecting and empowerment. The test of exogeneity was marginally significant (p = 0.08).

**Conclusions:**

We did not find evidence of a significant relationship between reproductive empowerment and desire to continue self-injecting. Though there are limitations to this secondary data analysis, we recommend future research investigate this relationship using the methodology demonstrated to address endogeneity inherent in answering this critical question about self-care interventions.

## Introduction

According to the World Health Organization (WHO), self-care interventions in family planning are a promising approach to improve reproductive health. Self-care is defined as “the ability of individuals, families and communities to promote health, prevent disease, maintain health and cope with illness and disability with or without the support of a health worker” [1]. Self-care spans a range of practices including self-awareness, self-testing, and self-management. Family planning self-care interventions, such as self-administration of contraceptive injectables, have the potential to reduce contraceptive unmet need by increasing access and coverage; to improve health equity by reaching new users; to ensure continuity of pregnancy protection during humanitarian emergencies; and to reduce costs to the health system. The WHO endorsed contraceptive self-injection with a “strong recommendation” in the *Consolidated Guidelines on Self-Care Interventions for Health* [2].

Contraceptive self-injection is available through a formulation of depot medroxyprogesterone acetate (DMPA) delivered subcutaneously (DMPA-SC) in a prefilled, auto-disabled Uniject™ injection system, known by the brand name Sayana® Press (Pfizer, New York, NY, USA). This easy-to-use injectable method is suitable for administration by community health workers and by clients themselves [3–9]. After being trained, a self-injector may be dispensed additional doses to take away for future use. DMPA-SC self-injection is gaining considerable traction such that the product is now available in at least 30 FP2030 countries and is approved by regulatory agencies in nearly 60 countries worldwide [10]. Forty-three countries have introduced or are currently introducing self-injection, of which 16 countries are in the process of scaling up self-injection, all in sub-Saharan Africa [11].

Self-care interventions, particularly related to family planning, have often been described to have benefits beyond health care, with statements that they can be empowering to users [12]. The WHO describes empowerment as a core principle of self-care*,* however, empowerment is a broad concept. One dimension of this concept related to contraception is reproductive empowerment. The International Center for Research on Women and MEASURE Evaluation define reproductive empowerment as:

“Both a transformative process and an outcome, whereby individuals expand their capacity to make informed decisions about their reproductive lives, amplify their ability to participate meaningfully in public and private discussions related to sexuality, reproductive health and fertility, and act on their preferences to achieve desired reproductive outcomes, free from violence, retribution or fear” [13].

While it is widely assumed that family planning self-care interventions are empowering to users, little evidence supports this claim [14]. The self-care guidelines highlight this gap, directing researchers to investigate the relationship between reproductive empowerment and self-care. Existence of this relationship remains a question and so too does its directionality. It is hypothesized that use of self-care will lead to higher levels of empowerment, however, it is also possible that those who are more empowered are more likely to adopt self-care strategies. The direction of the relationship is important to understand because equity is a key consideration for self-care, and it is critical that self-care is accessible to all who need or want these interventions, not only those who are empowered. In addition, unmeasured confounding can contribute to an endogeneity problem. This and the potential reversed causality between empowerment and use of self-care make this relationship challenging to study [15]. If not addressed, endogeneity can lead to incorrect assertions about causality.

Recently, we conducted a systematic review examining the relationship between self-care and empowerment and we found substantial gaps in the evidence base [12]. Most of the studies in the review focused on condom use. The review identified positive relationships between condom use self-efficacy and use of/intention to use condoms. However, with few studies pertaining to other methods and their low-quality evidence, strong conclusions about other self-care contraceptive strategies could not be drawn. Further, only one of the 37 studies in the review explored DMPA-SC self-injection. The review also noted that few studies meeting the eligibility criteria employed validated measures of reproductive empowerment. As such, there is a need for research to investigate the relationship between other contraceptive self-care methods and reproductive empowerment using rigorous methods.

In a recent study assessing the pilot introduction of DMPA-SC self-injection in Addis Ababa, Ethiopia, we had the opportunity to include questions from a validated measure of reproductive empowerment. We present an analysis of these data that aims to explore the relationship between reproductive empowerment and the use and acceptability of DMPA-SC self-injection among new self-injectors. We use instrumental variable techniques to mitigate the problem of endogeneity [15].

## Methods

### Setting

The self-injection pilot study took place across six high-volume public health facilities in Addis Ababa, Ethiopia, that were selected by the Ministry of Health to pilot rollout of DMPA-SC self-injection. Recent data indicate that 48% of married women of reproductive age in Addis Ababa currently use a modern method of family planning [16]. Of those using any contraceptive method, injectables are the most widely used, representing 34% of the method mix in the city [16]. According to the 2016 Ethiopia Demographic and Health Survey, 32% of women in Addis Ababa had completed secondary education or higher and 88% were literate [17].

### Study design

We used data from our recent study which aimed to generate information on the safety, acceptability, and feasibility of introduction and scale-up of DMPA-SC self-injection in select sub-cities in Addis Ababa, Ethiopia. This was achieved through a mixed-methods implementation research design, enrolling a prospective cohort of adult family planning users seeking injectable contraceptive services from public sector providers, who were followed-up over a three-month period.

### Data collection

Four-hundred participants were recruited between August 10-December 22, 2021. All family planning users at the pilot sites who were medically eligible and interested in DMPA-SC self-injection were invited by public health family planning providers to participate in the study, until the target sample size was reached. The target sample size of 400 was determined to be sufficient for assessing competency in DMPA-SC self-injection at three months, which was the primary outcome of the pilot study. Trained research assistants administered a tablet-based enrollment questionnaire to document participants’ sociodemographic characteristics and perceptions of self-injection prior to training. Providers then trained participants to self-administer DMPA-SC, who were invited to practice self-injection on a prosthetic, and then inject themselves. Providers assessed participants’ self-injection performance using a standardized observation form [6,9,18–20]. Those who demonstrated that they could competently self-inject were given one additional unit of DMPA-SC to take away with them to reinject three months later. These participants were eligible for follow-up which took place from November 15, 2021 to April 30, 2022. During the follow-up visit, the research assistants administered a tablet-based questionnaire to capture participants’ experience with self-injection training, 3-month self-injection, storage, disposal, and side effects, as well as their perceptions on acceptability of the method. Participants then demonstrated self-injection technique on a prosthetic while the research assistants assessed their performance using the standardized observation tool.

Reproductive empowerment was measured during both the enrollment and follow-up surveys using the validated Reproductive Empowerment Scale [21,22]. This scale was selected because it meets the three levels of agency outlined by the Reproductive Empowerment Framework [23]. Specifically, this study utilized the reproductive health decision-making sub-scale as this component is most closely linked to individual behavior that may be related to DMPA-SC self-injection. The sub-scale is comprised of four questions scored from 1-4 for a total maximum score of 16, with higher scores representing higher levels of reproductive empowerment. Two of the questions employ Likert scales (from strongly disagree to strongly agree) focusing on one’s ability to use contraception or refuse sex when one’s partner is in opposition. The other two questions ask about one’s current and preferred contraceptive decision-makers such that those responding with “my partner and myself jointly” receive the highest score, then those saying “myself”, then “my partner” and finally all other options [24]. The mean response score was imputed for participants with two or fewer missing responses. Participants with three or more missing responses needed for the subscale were excluded from analysis.

Acceptability of DMPA-SC self-injection was assessed through the following questions (1) uptake of self-injection (measured through participation in the study), (2) re-injection at 3-months (dichotomous, measured at 3-month follow-up visit), and (3) desire to continue using DMPA-SC self-injection in the future (dichotomous, measured at 3-month follow-up visit). Related concepts of comfort and confidence with self-injection were also measured over time. Comfort with self-injection was measured using a 4-point Likert scale (very nervous, somewhat nervous, at ease, very at ease) and confidence was measured with a 3-point Likert scale (very confident, somewhat confident, not very confident). Participants were asked to consider their comfort with self-injection prior to self-injection training (measured during the enrollment survey prior to self-injection training), after training, and after reinjection approximately 3-months after training (both assessed in the follow-up survey). Confidence about self-injection was with respect to self-injection at enrollment, reinjection 3-months later, and regarding future self-injection, all of which were asked about during follow-up.

### Data analysis

Descriptive analysis was performed to describe the characteristics of participants who completed a 3-month follow-up visit. We first assessed if there were changes in reproductive empowerment by performing a paired t-test to compare reproductive empowerment scores from enrollment to the 3-month follow-up visit. We performed this same paired t-test assessing changes in reproductive empowerment scores again among a restricted sample that only included participants who had already reinjected at the time of follow-up to explore if there was a difference for this subgroup. We then built a multivariable model to adjust for confounders and assess the factors related to the change in reproductive empowerment scores from baseline to endline. We used a data-driven approach to build the model, starting with separate regression analyses for each of the variables under consideration, with inclusion of baseline empowerment as the only confounding factor. Variables were considered for analysis based on theoretical plausibility that they may affect reproductive empowerment. Any variable with p < 0.1 in this analysis was considered for inclusion in the final model. To avoid introducing endogeneity problems, variables measured at the time of follow-up were not eligible for inclusion in the model, with one exception. We considered self-injection competency at follow-up because this indicator was measured through independent observation of injection technique and may not be affected by the same factors as our measure of reproductive empowerment at follow-up. The model included the baseline reproductive empowerment score as a covariate to control for incoming levels of reproductive empowerment. We assessed the models using the variable inflation factor to identify multicollinearity problems, and AIC and R-squared, dropping additional variables based on this model assessment.

We selected one measure of acceptability of DMPA-SC self-injection to assess the relationship with empowerment. For this analysis, we considered desire to continuing using DMPA-SC self-injection as our main outcome of interest as it more closely reflects future behavior intentions, and we hypothesized that higher empowerment would lead to better acceptability. The modeling approach described below could be done for other acceptability measures.

To explore the relationship between empowerment and the desire to continue using DMPA-SC self-injection, we built an instrumental variable probit model. As described above, empowerment and measures of acceptability may be affected by similar unmeasured factors or have a reversed causality relationship. For example, is self-injecting empowering and/or are those with higher levels of empowerment more likely to continue self-injecting? This potential endogeneity could result in biased estimates unless an appropriate methodology is followed. To address the potential endogeneity, we identified instrumental variables of endline reproductive empowerment and then applied a two-stage regression approach to predict desire for continuation with an instrument for empowerment, controlling for other variables. For this exploratory analysis, we used a data-driven approach to identify suitable instrumental variables by first conducting a bivariate regression model for any variable that could potentially be related to desire to continue using self-injection, among those collected, and then selecting those with a p-value < 0.1 for inclusion in the multivariable model. Variables were further selected based on measures of good fit including AIC and BIC. We compared multivariable models for our main outcome of desire to continue self-injecting and that of our potentially endogenous variable of endline reproductive empowerment. The model for endline reproductive empowerment was used rather than that of change in reproductive empowerment as we theorized that one’s level of reproductive empowerment at follow-up was more likely to be related to future use than the change in empowerment experienced over the last 3 months. However, the final models for both endline and change in reproductive empowerment resulted in the inclusion of the same factors (Supplemental material). Variables found to be associated with endline reproductive empowerment but not with desire to continue with DMPA-SC self-injection were selected as instruments.

To help explain the model results, we also explored changes in comfort and confidence to self-inject over time (not in relation to reproductive empowerment). We compared individual change in response to the question regarding how comfortable participants felt with self-injecting over time, among those who re-injected at 3-months. We also compared individual change in response to the question regarding how confident participants felt about self-injection over time, among those who re-injected at 3-months. We calculated the proportion of participants who shifted responses between time points and tested whether significant shifts in response categories occurred between time points using the Stuart-Maxwell marginal homogeneity test for symmetry. A visual depiction of these shifts is illustrated through Sankey diagrams. All analyses were conducted using Stata version 16 and final tests of associations with p < 0.05 were considered significant for two-sided comparisons [25].

### Ethics

This study was reviewed and approved by the Ethiopian Public Health Institute Institutional Review Board and PSI’s Research Ethics Board. All participants provided written informed consent to participate.

## Results

Among the 343 participants surveyed at follow-up, over 40% had not completed primary school and 30% were 18-24 years old at enrollment (Table 1). Most identified as Orthodox Christian and 50% reported working for payment within the last year. A minority disclosed they were using family planning discretely and few had prior self-injection experience. At the three-month follow-up visit, most participants (78%) wanted to continue self-injecting DMPA-SC.

**Table 1:** Descriptive characteristics of participants followed-up at 3 months (n = 343).

Participant characteristic	No (%)
**Health facility**	
Health facility 1	25 (7.3)
Health facility 2	71 (20.7)
Health facility 3	75 (21.9)
Health facility 4	70 (20.4)
Health facility 5	61 (17.8)
Health facility 6	41 (12)
**Age**, *mean (sd) [range]*	27.8 (5.3) [18-47]
**Educational level achieved**	
No education	27 (7.9)
Some Primary, but incomplete	116 (33.8)
Completed Primary	39 (11.4)
Some Secondary, but incomplete	50 (14.6)
Completed Secondary	56 (16.3)
Some Post-secondary, but incomplete	19 (5.5)
Completed Post-secondary	36 (10.5)
**Attended postsecondary education**	
No	288 (84)
Yes	55 (16)
**Religion**	
Orthodox Christian	238 (69.4)
Protestant	47 (13.7)
Catholic	3 (0.9)
Muslim	55 (16)
**Employed in last year**	
No	172 (50.2)
Yes	171 (49.9)
**Covert user status**	
Not a covert user	309 (90.1)
Covert user	34 (9.9)
**Prior self-injection experience**	
No	314 (91.6)
Yes	24 (7)
Don’t know	5 (1.5)
**Injected by follow-up**	
No	104 (30.3)
Yes	239 (69.7)
**Desire to continue method**	
No	66 (19.2)
Yes	267 (77.8)
Don’t know	10 (2.9)
**Reproductive empowerment score at enrollment**	
*Mean (sd) [range]*	12.32 (2) [3–16]
**Reproductive empowerment score at follow-up**	
*Mean (sd) [range]*	12.28 (1.7) [3.3-16]
**Change in reproductive empowerment score**	
*Mean (sd) [range]*	-0.04 (2.2) [-7.7-11]

Sd =  standard deviation

### Reproductive empowerment and desire to continue using DMPA-SC self-injection

Reproductive empowerment scores were high both at baseline and endline (about 12 out of 16 points on the sub-scale for both, Table 1). There was no statistically significant difference in reproductive empowerment scores from enrollment to follow-up (n = 343; p = 0.76). Among the restricted sample of those who reinjected by the 3-month follow-up visit (n = 239), there was similarly no significant difference in reproductive empowerment scores over time (p = 0.77).

The final model of the change in reproductive empowerment (Table 2) included postsecondary education, employment, health facility, and religion, adjusting for baseline empowerment. Baseline reproductive empowerment scores were strongly independently associated with the change in reproductive empowerment in this model. For each 1-point increase in baseline reproductive empowerment, there was a 0.77-point decrease in the change in scores, such that participants with higher initial reproductive empowerment scores experienced less of a change in their score.

**Table 2:** Factors associated with change in reproductive empowerment score (n = 340).

Characteristic	Adjusted for baseline reproductive empowerment only Coefficient (95% CI)	Final model Coefficient (95% CI)
**Postsecondary education**	0.59 (0.12-1.05)*	0.53 (0.04-1.02)*
**Employed**	0.38 (0.04-0.73)*	0.35 (0-0.69)*
**Health facility (ref=HF 1)**	**	**
Health facility 2	-0.14 (-0.87-0.59)	-0.18 (-0.9-0.54)
Health facility 3	0.72 (-0.01-1.44)	0.71 (0-1.43)
Health facility 4	-0.13 (-0.86-0.6)	-0.2 (-0.91-0.52)
Health facility 5	-0.02 (-0.76-0.73)	-0.35 (-1.11-0.4)
Health facility 6	0.66 (-0.13-1.45)	0.62 (-0.16-1.4)
**Religion** *(ref = Orthodox Christian)*	*	*
Protestant	-0.58 (-1.08--0.07)*	-0.65 (-1.15--0.15)*
Muslim	-0.37 (-0.85-0.12)	-0.46 (-0.95-0.03)
**Reproductive empowerment score at enrollment**	N/A	-0.77 (-0.85--0.68)**
**Age**	-0.02 (-0.05-0.02)	
**Covert user**	0.16 (-0.42-0.74)	

*p < 0.05,

**p < 0.01; N/A =  not applicable; CI =  confidence interval; ref =  reference group.

Attendance of postsecondary education and working within the last year were significantly associated with increases in reproductive empowerment, after adjusting for other factors. Religion was significantly independently associated with the change in reproductive empowerment, whereby participants identifying as Protestant had a 0.65-point lower change in reproductive empowerment score than those identifying as Orthodox Christian. There were only three participants who identified as Catholic. Due to issues of small cell size and since we considered it inappropriate to combine these participants into another category of religion, they (<1% of sample) were excluded from this analysis. Enrollment health facility was related to the change in reproductive empowerment however no one health facility was significantly different from the reference facility. The model explained 51% of the variability in the change in reproductive empowerment scores.

To develop the instrumental variable probit model for desire to continue using DMPA-SC self-injection, we first modeled endline reproductive empowerment and desire for continuation separately. We compared the variables included in each of the models of best fit. Variables significantly related to endline reproductive empowerment that were not significantly associated with desire to continue DMPA-SC were identified as instruments. These included religion, employment status and postsecondary school attendance. This is consistent with the results of the model for change in empowerment in Table 2. The final model did not identify a significant relationship between desire to continue using DMPA-SC self-injection and reproductive empowerment either at baseline or at follow-up using the instruments, after adjusting for other variables (Table 3). Desire to continue the method was significantly associated with participants’ health facility at enrollment and their experience of side effects, when accounting for other factors. Compared to participants who did not experience any side effects, those who did experience them and reported increasing levels of interference in their everyday life, were significantly less interested in continuing to use DMPA-SC self-injection in the future. The Wald test of exogeneity was not significant at the p < 0.05 level, indicating there is insufficient information in the sample to conclude that there is a clear endogeneity problem, though it was marginally significant (p = 0.085).

**Table 3:** Factors associated with desire to continue using DMPA-SC self-injection (n = 325).

Characteristic	Coefficient (95% CI)	p-value
**Health facility**		
Health facility 2	1.13 (0.36-1.91)	0.004
Health facility 3	0.64 (-0.27-1.54)	0.166
Health facility 4	0.38 (-0.28-1.04)	0.261
Health facility 5	1.47 (0.47-2.46)	0.004
Health facility 6	0.57 (-0.32-1.47)	0.211
**Interference of side effects** *(ref: no side effects experienced)*		
Not at all	-0.31 (-0.8-0.17)	0.203
Very little	-0.96 (-1.74-0.19)	0.014
Little	-1.32 (-2.22-0.42)	0.004
Moderate	-1.74 (-2.85-0.62)	0.002
Very much	-2.83 (-4.23-1.43)	0.000
**Competence at follow-up**	0.29 (-0.3-0.87)	0.335
**Reproductive empowerment score at baseline**	-0.1 (-0.23-0.04)	0.160
**Reproductive empowerment at follow-up** *(instrumented)*	0.32 (-0.05-0.69)	0.086

Instruments: religion, employment status, postsecondary education.

Wald test of exogeneity (corr = 0): chi2(1) = 2.97 Prob> chi2 = 0.0849.

### Comfort and confidence to self-inject over time

**Fig 1:**
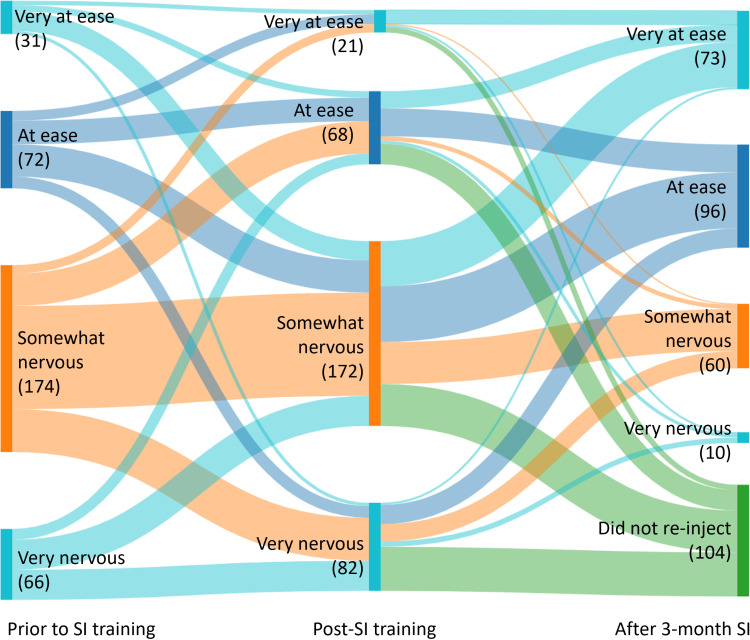
Comfort with DMPA-SC self-injection across time points (n = 343). *SI=self-injection.

About half of participants felt somewhat nervous about DMPA-SC self-injection at enrollment, prior to receiving self-injection training (174/343, 51%; Fig 1). There was no significant change in individual reporting of comfort level with self-injection from pre- to post-training (p = 0.17). However, there was a significant shift in participants’ level of comfort reported at the 3-month follow-up as compared to their feelings immediately after training, among those who reinjected (n = 239, p < 0.001). Among the 133 participants who said they felt somewhat nervous immediately after training, only 29% still felt somewhat nervous after their 3-month reinjection while 71% said they then felt at ease or very at ease. Similarly, only 12% of the 82 participants who felt very nervous post-training continued to feel very nervous after reinjection, while 81% of those who said they were very at ease remained at ease. Those who did not reinject by the time of the follow-up visit were included in Fig 1 to illustrate the pathways of all 343 participants. However, the test for comparing the shifts between the two time points only considers the 239 who reinjected (70% of the 343 participants follow-up at 3-months). The majority of those who did not reinject had felt very or somewhat nervous about self-injection. It should be noted however that some participants who had not reinjected at the time of follow-up said they still planned to do so. At the time of follow-up, 71% of respondents (169/239) reported feeling at ease or very at ease with self-injection. To see the results of Fig 1 in table format see Additional File 1.

**Fig 2:**
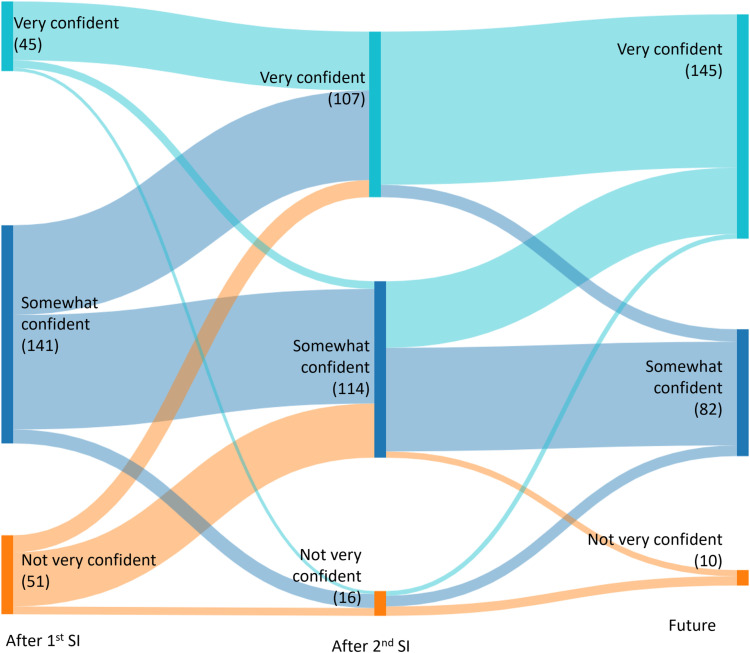
Confidence to self-inject DMPA-SC over time (n = 237).

The changes in confidence over time followed similar trends (Fig 2 and Additional File 1). All three questions about confidence were included in the follow-up survey but asked participants to reflect on different experiences – their confidence during their first injection, during their second injection at 3-months and about future self-injection. Most participants (59%) said they felt somewhat confident about injecting their first time at the health facility. There was a significant shift in participants’ reported confidence levels from this first injection to their second injection such that respondents indicated higher levels of confidence (p < 0.001). For example, only 10% of those who initially said they felt *not very confident* continued to feel this way after reinjection, while the other 90% said they now felt somewhat or very confident. There continued to be a significant shift towards higher confidence levels from one’s reinjection to one’s perception about future injection (p < 0.001). 93% of participants who felt very confident at reinjection also anticipated feeling very confident about future self-injection. Of those who said they were somewhat confident about their 3-month injection, 59% continued to feel this way while 38% felt very confident about future use. Overall, 61% of respondents said they were very confident about their ability to self-inject in the future.

## Discussion

As family planning self-care interventions such as contraceptive self-injection become increasingly available around the world, there is an urgent need to understand if these interventions are further widening access gaps between those who have greater versus fewer resources—including power to achieve their desired reproductive outcomes. It is also important to understand if these interventions can increase users’ reproductive empowerment which may offer benefits beyond pregnancy prevention [14]. However, studying this important, but potentially reversed causal relationship is complicated by the potential for endogeneity requiring researchers to take additional steps during data analysis to avoid making incorrect causal statements [15]. Using longitudinal data from a recent pilot study of DMPA-SC self-injection, we explored the relationship between reproductive empowerment and the desire to continue using DMPA-SC self-injection. These constructs are theoretically endogenous, and their relationship cannot be assessed through traditional association analyses. We used a two-stage regression approach to predict desire for continuation with an instrument for empowerment, controlling for other variables.

We found that participants in this pilot study had high reproductive empowerment scores at both enrollment and the 3-month follow-up visit, and we observed nonsignificant differences in scores over time. The study population may have been highly empowered to begin with (at least according to the measure of empowerment we used) because they all agreed to join our study and try self-injection—a new method being offered in their communities. Since scores were high to begin with there was not much room in the scale for the scores to increase over a 3-month period and this “ceiling effect” likely prevented us from observing differences over time. At least in this highly empowered population, we did not find evidence of self-care use affecting empowerment. This analysis should be replicated with a population with lower initial empowerment. Further, future studies should examine this relationship for those who are eligible for but decide not to try self-injection.

We explored models for change in empowerment from baseline as well as for endline empowerment to identify suitable instrumental variables. The variables found to be associated with endline reproductive empowerment but not with desire to continue with DMPA-SC self-injection were selected as instruments. These included religion, employment status and postsecondary school attendance, and are consistent with previous research that did not find women’s sociodemographic factors associated with the risk of discontinuation of DMPA-SC [5].

Our final model did not identify a significant relationship between reproductive empowerment, either at baseline or at follow-up using the instruments, and desire to continue using DMPA-SC self-injection. While advocates of self-care describe self-injection as empowering, our findings do not support this conclusion. At the same time, we only measured a change in reproductive empowerment over a short time frame (3-months, only one reinjection) but perhaps changes take longer to occur. This warrants further research with longer follow-up. Also, we specifically explored reproductive empowerment and measured it through a specific measure [21]. There are several scales available that measure reproductive empowerment-related concepts which cover different domains [23]. If we had used another measure of reproductive empowerment, we might have found a different outcome. Also, as noted in the background section, empowerment is a broad concept with many related constructs. It is possible that self-injection, and perhaps self-care more broadly, are not related to reproductive empowerment but rather other types of empowerment. While the test of exogeneity did not meet our prespecified threshold for significance, it is marginally significant and the constructs theoretically endogenous and as such, we still recommend future research investigate this relationship using appropriate methodology beyond single equation models. In addition, measurement of latent constructs poses additional challenges. Other measures of acceptability of self-injection may be more clearly endogenous. In either case, previous research examining simple associations are methodologically flawed and researchers should always approach this question first with an analysis addressing endogeneity. After testing and confirming no endogeneity in a particular analysis, researchers could then modify their models.

The factors that we found significantly associated with desire to continue self-injection—participants’ experience of side effects and the health facility where they were enrolled into the study—are consistent with previous studies [26–28]. Compared to participants who did not experience any side effects, those who did experience them and reported increasing levels of interference in their everyday life, were significantly less interested in continuing to use DMPA-SC self-injection in the future. The association with health facility may be related to differences in the (unmeasured) characteristics of participants attending the different health facilities or may be related to variations in provider counseling and training on DMPA-SC self-injection. This site-level effect warrants further investigation to ensure that counseling and training are high-quality and empathy-based across all health facilities.

Like other studies, we found that comfort and confidence to self-inject increased over time [7,29]. Interestingly, we found no change in comfort level from pre- to post-training, but we did see a significant increase in participants’ comfort after re-injection 3-months after training. It appears that users begin to feel more comfortable with this method after having injected themselves once on their own. This suggests that it is important for providers to focus on helping adopters through the first injection, representing a key window of opportunity that may support continued self-injection. We found that participants’ confidence significantly increased over time from the first injection to the 3-month re-injection, and towards future self-injections. Only 4% of participants who re-injected at 3-months were not very confident in their ability to self-inject in the future. These results indicate that there may be further gains in constructs related to empowerment over a longer period of time. Exploring measures of self-confidence and self-efficacy may be of interest in future research on this topic.

We recommend that other researchers who wish to examine the relationship between use of self-injection, and reproductive empowerment should employ this two-stage regression approach with instrumental variables or similar approaches. These types of constructs are theoretically endogenous, especially when they are measured at the same time point. Endogeneity also arises when data only include one study group, such as DMPA-SC self-injection users without comparators. We also advise the use of a long follow-up period, and a validated reproductive empowerment scale that yields more variability in the study population.

## Limitations

As a secondary analysis, we were limited by the study design and data that were collected to answer the primary research question. In particular, the short follow-up period may have restricted our ability to assess changes in reproductive empowerment which may develop over a longer period. The two main outcomes in this analysis are conceptually endogenous, as described in the introduction. Our analysis attempted to address the endogeneity concern, but it is affected by the data (measurement and design) as well as the potential for some misspecification (important confounders, appropriate instruments). Other study designs and inclusion of different subgroups could mitigate the endogeneity concerns by controlling for who chooses to participate in self-care, by design. On the other hand, our analysis is strengthened by the use of longitudinal data, which is rarely available. Further, our analysis uses robust methods to appropriately disentangle the endogeneity in our outcomes, offering more valid conclusions than naive regression analysis. Both better data and appropriate modelling addressing the endogeneity of these outcomes, are needed. Importantly, endogeneity should not be ignored.

Another limitation of this study is related to some of our outcome measures. The reproductive empowerment scores among this study sample were already high at baseline, leading to a potential ceiling effect. With a more variable distribution, we may have found different results, and therefore we encourage other researchers to explore this question with different populations and/or using different measures of reproductive empowerment. Also, our measures of comfort with self-injection post-training and after re-injection 3-months later were measured at the same time, during the 3-month follow up visit. However, it is reassuring that despite the two measures being taken at the same time, the responses to these questions varied which is an indication that the participants were engaged during the survey and increases our confidence in the validity of their responses.

## Conclusions

To our knowledge, this is the first analysis examining the relationship between reproductive empowerment and a contraceptive self-care intervention that used a modelling technique to better address the endogeneity problem that is rooted in the nature of these constructs. We also attempted to assess the directionality between the two constructs—a pressing question in the family planning self-care space. We did this through two models: the first assessed the change in empowerment after participants’ first self-injection and the second assessed the effect of empowerment on potential future use self-injection. Though the data we had available to us in this secondary data analysis had limitations, through this analysis we shine a light on the serious issue of endogeneity inherent in answering this question and offer a strategy for how to appropriately answer it in future studies.

## Supporting information

S1 File Change in comfort and confidence to self-inject over time. (DOCX)
