# Supplementary material for: Examining the relationship between reproductive empowerment and contraceptive self-injection: Tackling the endogeneity problem
Source: PLoS One. 2025 Feb 24;20(2):e0319330. doi: 10.1371/journal.pone.0319330 (PMC11849906; doi:10.1371/journal.pone.0319330)
Supplement: S1 File — (DOCX) [file pone.0319330.s001.docx]

# Supplementary materials

Table 1. Change in comfort to self-inject DMPA-SC over time

1. Pre- to post-self-injection training (n=343, p=0.17)

| Comfort with self-injection prior to training | Comfort with self-injection immediately after training | | | | |
| --- | --- | --- | --- | --- | --- |
|  | Very at ease | At ease | Somewhat nervous | Nervous | Total |
| Very at ease | 4 (13%) | 6 (19%) | 18 (58%) | 3 (10%) | 31 |
| At ease | 9 (13%) | 22 (31%) | 30 (42%) | 11 (15%) | 72 |
| Somewhat nervous | 8 (5%) | 30 (17%) | 96 (55%) | 40 (23%) | 174 |
| Nervous | 0 (0%) | 10 (15%) | 28 (42%) | 28 (42%) | 66 |
| Total | 21 | 68 | 172 | 82 | 343 |

1. Post-self-injection training to post-3-month injection (n=239, p<0.001)

| Comfort with self-injection immediately after training | Comfort with self-injection immediately after reinjection | | | | |
| --- | --- | --- | --- | --- | --- |
|  | Very at ease | At ease | Somewhat nervous | Nervous | Total |
| Very at ease | 13 (81%) | 0 (0%) | 1 (6%) | 2 (13%) | 16 |
| At ease | 16 (33%) | 26 (53%) | 4 (8%) | 3 (6%) | 49 |
| Somewhat nervous | 42 (32%) | 52 (39%) | 39 (29%) | 0 (0%) | 133 |
| Nervous | 2 (5%) | 18 (44%) | 16 (39%) | 5 (12%) | 41 |
| Total | 73 | 96 | 60 | 10 | 239 |

Table 2. Change in confidence to self-inject over time

1. First to second injection (n=237, p<0.001)

| Confidence to self-inject at enrollment  (1^st^ injection) | Confidence to self-inject 3-months later (2^nd^ injection) | | | |
| --- | --- | --- | --- | --- |
|  | Very confident | Somewhat confident | Not very confident | Total |
| Very confident | 38 (84%) | 5 (11%) | 2 (4%) | 45 |
| Somewhat confident | 58 (41%) | 74 (52%) | 9 (6%) | 141 |
| Not very confident | 11 (22%) | 35 (69%) | 5 (10%) | 51 |
| Total | 107 | 114 | 16 | 237 |

1. Second injection to future (n=237, p<0.001)

| Confidence to self-inject 3-months later (2^nd^ injection) | Confidence to self-inject in the future | | | |
| --- | --- | --- | --- | --- |
|  | Very confident | Somewhat confident | Not very confident | Total |
| Very confident | 99 (93%) | 8 (7%) | 0 (0%) | 107 |
| Somewhat confident | 43 (38%) | 67 (59%) | 4 (4%) | 114 |
| Not very confident | 3 (19%) | 7 (44%) | 6 (38%) | 16 |
| Total | 145 | 82 | 10 | 237 |
